# Tumor suppressors revival in CLL

**DOI:** 10.18632/aging.101258

**Published:** 2017-06-26

**Authors:** Giovanna Carrà, Riccardo Taulli, Alessandro Morotti

**Affiliations:** Department of Clinical and Biological Sciences, University of Turin, 10043 Turin, Italy

**Keywords:** Chronic Lymphocytic Leukemia, tumor suppressors, USP7, PTEN, p53, FOXO

Since the 2001 FDA approval of the BCR-ABL tyrosine kinase inhibitor, Imatinib, as the standard of treatment for Chronic Myeloid Leukemia (CML), these last 16 years have clearly showed the raise and the fall of strategies to target oncogenes in cancer. Beside the successful story of Imatinib in CML, no other drugs able to target oncogenes at the bench have shown such impressive results at the bedside. While various compounds have indeed clearly prompted themselves as effective drugs with promising clinical results, still the cure for cancer, meaning complete eradication, remains a mirage with these inhibitors.

Conversely, murine models have proven that the re-establishment of tumor suppressors in cancer remains the unique strong strategy to obtain cancer eradication: in a very simple, yet dramatically effective manner, the re-expression of p53 in various cancer models have indeed been associated with cancer exhaustion [1].

For many years, the strategies to target tumor suppressors have been neglected from drugs cocktails. The idea that tumor suppressors are involved in tumorigenesis through genetic impairments of both alleles and the lack of strategies to restore these genes in cancer cells have wiped out all the promises in targeting these genes. Yet, the mechanisms of tumor suppressors impairment in cancer have changed: it is now clear that even wild-type tumor suppressors can play an essential role in tumorigenesis, when functionally inhibited [2]. Mechanisms that promote tumor suppressors delocalization, degradation and/or inactivation inevitably result in tumor suppressors inhibition. The identification of tumors that depend on functionally inactive tumor suppressors is of extraordinary importance because these tumors can potentially benefit from therapies designed to restore the function of the inactive tumor suppressors.

Very recently, we have demonstrated that the de-ubiquitinase USP7, also known as HAUSP, is aberrantly expressed and active in Chronic Lymphocytic Leukemia (CLL) [3]. Besides being one of the hundreds of differentially expressed genes in a cancer, USP7 has the privilege of controlling the expression, localization and function of three major tumor suppressors: PTEN [4, 5], p53 [6] and FOXO [7].

In this work, we demonstrated that CLL is characterized by an increased USP7 expression, through miRNA deregulation, and by aberrant USP7 regulation through Casein Kinase II. Consequently, USP7 was shown to promote PTEN delocalization from the nucleus with consequence loss of part of its tumor suppressive functions. Conversely, USP7 inhibitor restores PTEN nuclear pool with re-establishment of its tumor suppressive functions. In this work, we focused on the ability of USP7 to modulate PTEN in a p53 null scenario, which remains the most challenging battlefield for CLL therapy. However, it should be noted that USP7 is also well known to module p53 protein levels, as well [6]. USP7 promotes mdm2 de-ubiquitination, which in turn modulates p53 protein degradation. High levels of USP7 activation can indeed affect the mdm2/p53 network with potentially intriguing consequences on p53 protein levels and functional regulation. Similarly, USP7 was shown to module the mono-ubiquitination of FOXOs, very known tumor suppressors able to control cellular proliferation [7]. USP7 favors FOXOs nuclear exclusion and inactivation. While we did not investigate FOXO cellular compartmentalization and p53 protein levels in CLL, it could be speculated that high levels of USP7 may also affects FOXO localization and p53 protein levels in CLL.

Our published observation that USP7-PTEN is a targetable network in CLL [3], and the above speculations that USP7 may also affect p53 and FOXOs in the CLL context, attribute to USP7 a potential pivotal role in the functional regulation of three major tumor suppressors in CLL. Therefore, USP7 inhibitors may represent strong apoptotic inducers in CLL through: *i*) the reactivation of wild-type PTEN and, potentially, p53 and FOXO; *ii*) by-passing the resistance mediated by mutations/deletions of one of these tumor suppressors through the reactivation of the others, as we observed in the presence of p53 mutations [3].

In summary, our data clearly indicate that the characterization of molecular circuits involved in the control of oncosuppressor stability, localization and activity is critical to develop novel therapeutic strategies aimed at re-activating oncosuppressor functions. Thus, it is now advisable that USP7 inhibitors will be included among the drugs to be further investigated for their ability to positively modulate oncosuppressor regulatory networks in cancer.
